# The Body Action Coding System II: muscle activations during the perception and expression of emotion

**DOI:** 10.3389/fnbeh.2014.00330

**Published:** 2014-09-23

**Authors:** Elisabeth M. J. Huis In ‘t Veld, Geert J. M. van Boxtel, Beatrice de Gelder

**Affiliations:** ^1^Brain and Emotion Laboratory, Department of Medical and Clinical Psychology, Tilburg UniversityTilburg, Netherlands; ^2^Department of Cognitive Neuropsychology, Tilburg UniversityTilburg, Netherlands; ^3^Brain and Emotion Laboratory, Faculty of Psychology and Neuroscience, Maastricht UniversityMaastricht, Netherlands

**Keywords:** surface EMG, body, emotion, coding, movement, muscles, perception, expression

## Abstract

Research into the expression and perception of emotions has mostly focused on facial expressions. Recently, body postures have become increasingly important in research, but knowledge on muscle activity during the perception or expression of emotion is lacking. The current study continues the development of a Body Action Coding System (BACS), which was initiated in a previous study, and described the involvement of muscles in the neck, shoulders and arms during expression of fear and anger. The current study expands the BACS by assessing the activity patterns of three additional muscles. Surface electromyography of muscles in the neck (upper trapezius descendens), forearms (extensor carpi ulnaris), lower back (erector spinae longissimus) and calves (peroneus longus) were measured during active expression and passive viewing of fearful and angry body expressions. The muscles in the forearm were strongly active for anger expression and to a lesser extent for fear expression. In contrast, muscles in the calves were recruited slightly more for fearful expressions. It was also found that muscles automatically responded to the perception of emotion, without any overt movement. The observer's forearms responded to the perception of fear, while the muscles used for leaning backwards were activated when faced with an angry adversary. Lastly, the calf responded immediately when a fearful person was seen, but responded slower to anger. There is increasing interest in developing systems that are able to create or recognize emotional body language for the development of avatars, robots, and online environments. To that end, multiple coding systems have been developed that can either interpret or create bodily expressions based on static postures, motion capture data or videos. However, the BACS is the first coding system based on muscle activity.

## Introduction

Faces, bodies, and voices are the major sources of social and emotional information. There is a vast amount of research on how humans perceive and express facial expressions. Some of this resulted in the creation of the Facial Action Coding System (FACS) which extensively describes which muscles are recruited for emotional expressions (Ekman and Friesen, 1978). Using the FACS and electromyography recordings (EMG), many studies have examined conscious and unconscious facial responses to emotional faces (Dimberg, 1990; see Hess and Fischer, 2013 for a review). In the last decade it has become increasingly clear that bodily expressions are an equally valid means of communicating emotional information (de Gelder et al., 2004; de Gelder, 2009). Bodily expressions, more so than facial expressions, quickly activate cortical networks involved in action preparation, action understanding and biological motion (Kret et al., 2011; de Gelder, 2013), and especially for emotions that are negative or threatening (de Gelder et al., 2004). For example, the perception of an angry or fearful person activates a network in the brain that facilitates the perception and execution of action, such as the (pre)motor areas and the cerebellum (Grezes et al., 2007; Pichon et al., 2008, 2009, 2012). This corroborates the idea that in daily life, expressing emotion with the body is an automatic, reflex-like behavior that is often triggered as soon as a response is required to something in the environment (Frijda, 2010). In line with this theory, transcranial magnetic stimulation (TMS) studies found motor facilitation of those muscles that were used in an observed movement (Fadiga et al., 2005), but also increased corticospinal excitability in response to fearful faces (Schutter et al., 2008) and emotional bodily expressions (Borgomaneri et al., 2012). In addition, Coombes et al. (2006, 2009) found increased force production and motor evoked potentials of finger and wrist extensors following a negative stimulus. Studies in a more clinical setting have found that psychological factors, such as fear of pain, influence the activation patterns of muscles in the back in chronic lower back pain patients (Watson et al., 1997; Geisser et al., 2004). Aditionally, muscles in the legs have a different muscle tone if one imagines the self in a painful situation (Lelard et al., 2013). These results inspired the question of whether automatic and covert muscle responses to emotion are limited to the face, or whether such activations might also be found in body muscles. To answer that question, the role of muscles in the neck, shoulders and arms during the expression and perception of angry and fearful emotions were recently assessed using EMG (Huis in `t Veld et al., 2014). It was found that it is indeed possible to measure covert responses of muscles in the body. Distinctly different response patterns of muscles in the neck and arms to the perception of fearful and as opposed to angry bodily emotions were found. However, to assess the underlying mechanisms of these activations, it is necessary to know what role these muscles play in the execution of emotion. The study by Huis in `t Veld et al. (2014) was the starting point of the Body Action Coding System (BACS), with the aim of creating a system that not only describes which muscles are used for emotional bodily expressions, but also which muscles respond covertly to the perception of emotion.

Understanding the role of the body in emotional expression is becoming increasingly important, with interest in developing systems for gaming (Savva and Bianchi-Berthouze, 2012; Zacharatos, 2013), robots (Castellano et al., 2013; Mccoll and Nejat, 2014), touchscreen interfaces (Gao et al., 2012) or even teaching (Mayer and Dapra, 2012), and tools for public speaking (Nguyen et al., 2013). To that aim, multiple coding systems have been developed that can either interpret or create bodily expressions, based on static postures, motion capture data or videos (see Zeng et al., 2009; Calvo and D'mello, 2010; Karg et al., 2013; Kleinsmith and Bianchi-Berthouze, 2013 for some extensive reviews). However, no system exists yet based on muscle involvement and electromyography (EMG) measurements. If such a system were available, it would enable research on automatic responses without overt movement and build natural emotional expressions based on biologically valid movement data. The current study continues the work on the BACS by firstly assessing the role of muscles in the lower back, forearms and calves involved in the expression of fear and anger, and secondly, determine whether it is possible to measure covert responses in these muscles during the passive viewing of emotion.

## Materials and methods

The stimuli described below, the experimental procedure, EMG data acquisition, processing procedures and statistical analyses are similar to what is reported in the previously mentioned BACS I article (Huis in `t Veld et al., 2014).

### Participants

Forty-eight undergraduates of Tilburg University participated in exchange for course credit. Participants read and signed an informed ethical consent form and completed a screening form to assess physical, psychological, and neurological health. The study was approved by the Maastricht University ethical committee. The following participants were excluded from analysis: one subject suffered from hypermobility, one from fibromyalgia, four used medication, three were left-handed, and one did not adhere to the instructions. The data for three participants was of overall low quality and sessions for five participants were aborted by the researcher; two felt uncomfortable standing still (which made them dizzy), two felt uncomfortable in the electrically shielded room (indicating it was oppressive), and one felt unwell. Three participants failed to adhere to the dress code which prevented the measurements of the calf muscles, but all other muscles were measured. The sample therefore consisted of 30 healthy right-handed individuals between 18 and 24 year old, 12 males (*M* = 21.2, *SD* = 2.2) and 18 females (*M* = 19.5, *SD* = 2.2) with normal or corrected-to-normal vision.

### Stimulus materials and procedure

**Figure 1:**
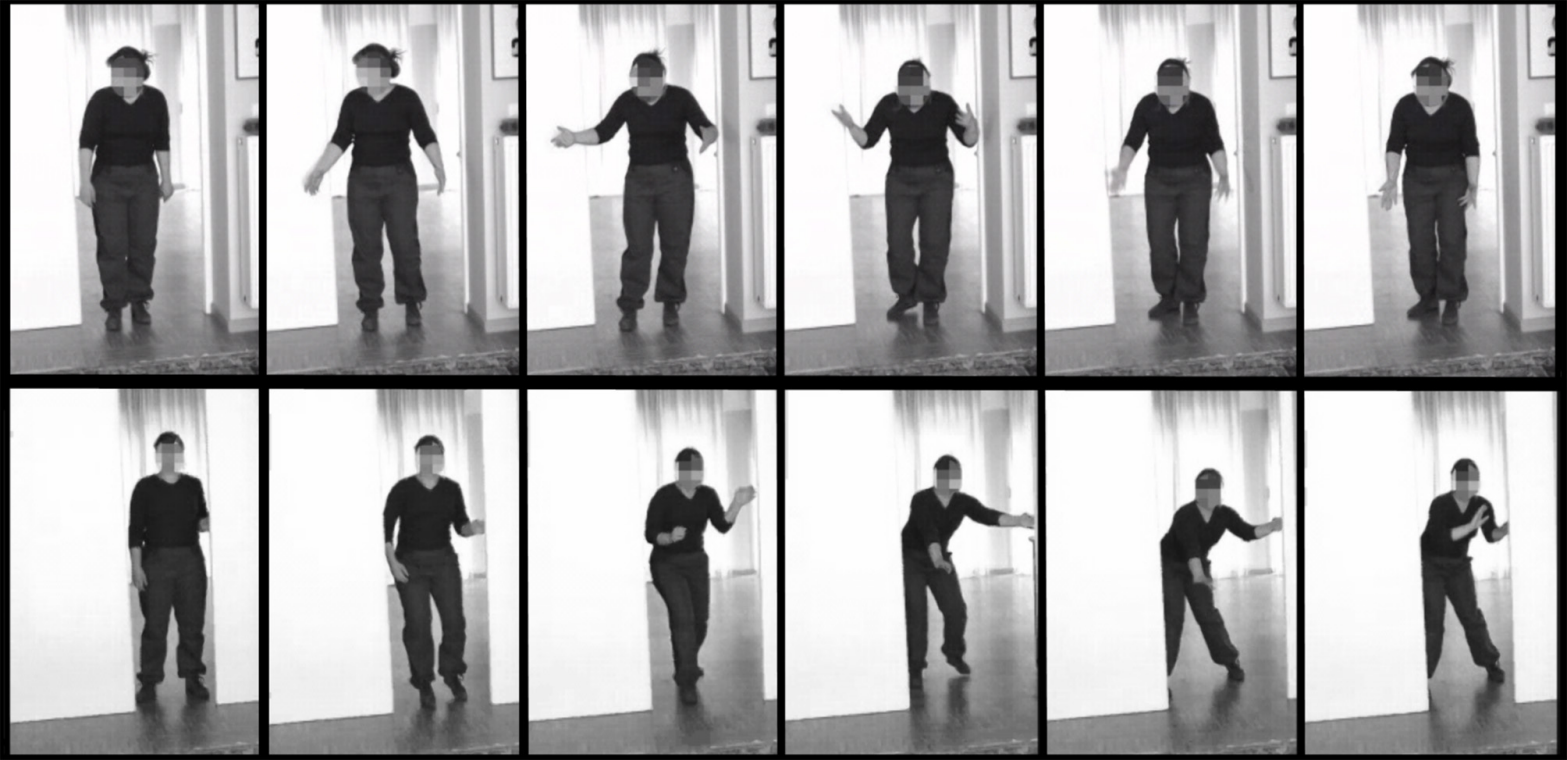
**Stimulus examples**. Still frames from an angry (upper) and fearful (lower) stimulus video.

The experiments consisted of two emotion conditions, Fear and Anger. Twenty-four videos of 3000 ms were used, in which an actor opens a door followed by a fearful (12 videos) or angry (12 videos) reaction (see Figure 1). These stimuli have been used in other studies (Grezes et al., 2007; Pichon et al., 2008, 2009, 2012) and are well-recognized and controlled for movement and emotional intensity. The face of the actor was blurred. The videos were projected life-size on the wall, in front of the participant who was standing upright. In total there were 72 randomly presented trials, 36 for the Anger condition and 36 for the Fear condition, with an inter-trial interval (ITI) between 9 and 11 s. During the ITI a black screen with a white fixation cross at chest height of the stimulus was shown. The experiment was divided into 2 blocks of 36 trials with a break in between. The same procedure was used in both experiments.

### Experiment 1: passive viewing

The participants were instructed to view all the videos while maintaining an upright posture with the head facing forward, feet positioned 20–30 cm apart, squared but relaxed shoulders and arms hanging loosely next to the body. They were asked to stand as still as possible while keeping a relaxed stance, and to minimize unnecessary movements, such as moving the head, shifting stance, or tugging at hair or clothing.

### Experiment 2: imitation

**Figure 2:**
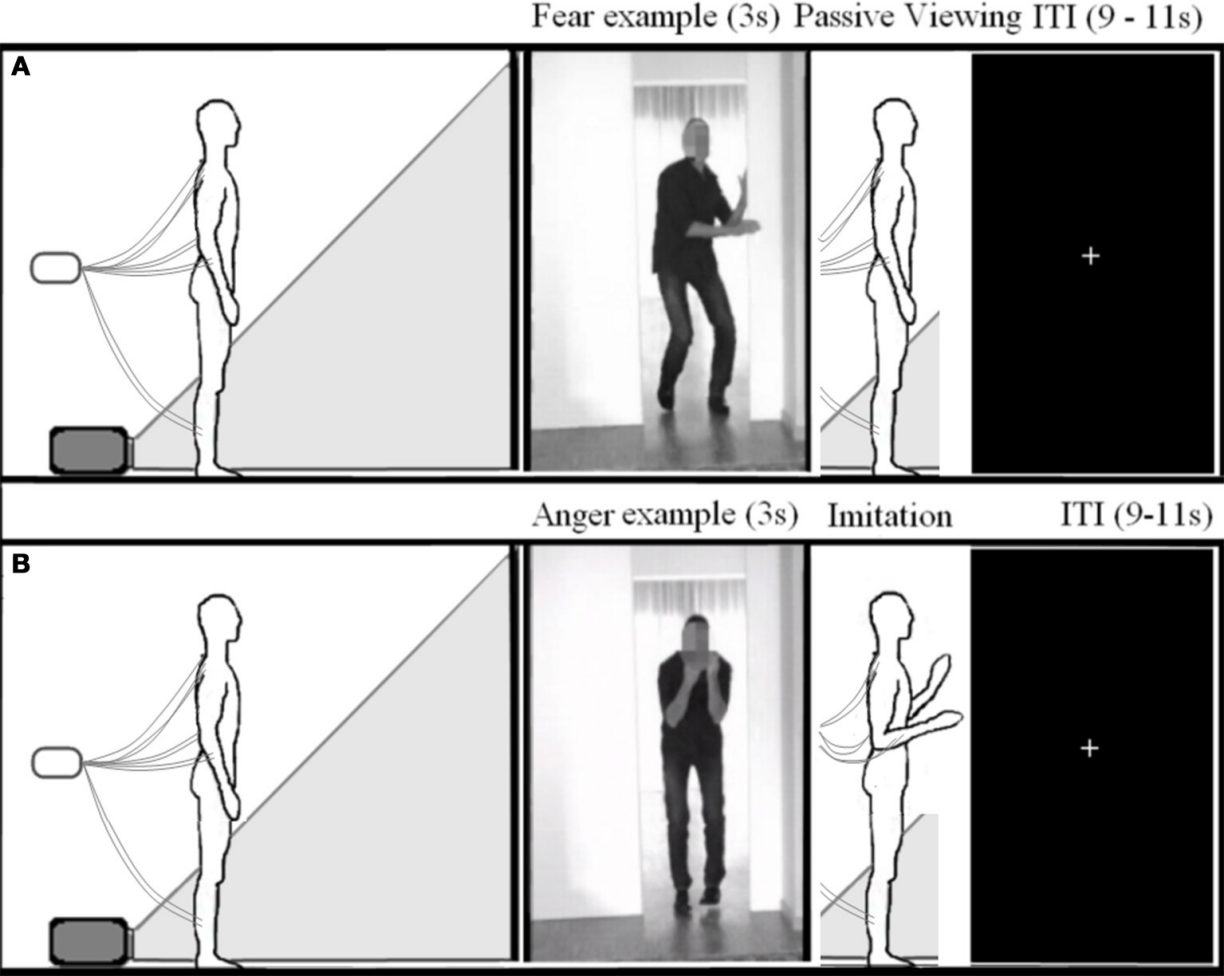
**Experimental setup**. Schematic overview of the experimental setup for experiment 1 **(A)** and 2 **(B)**.

The participants were told that they would see the same videos as in experiment 1 and instructed to mimic the emotional reaction of the actor. They were urged to do this as convincingly as possible using their whole body. The subjects first viewed the whole video adopting the same stance as in experiment 1, and after the offset of each movie, imitated the emotional movement of the actor and then returned to their starting position and posture. This was first practiced with the experimenter to ensure the subjects understood the procedure. (see Figure 2). Experiment 2 always followed experiment 1. The order of the experiments was not counterbalanced, in order to prevent habituation to the videos and to keep the participants naïve as to the purpose of the study during the passive viewing experiment.

### Electrophysiological recordings and analyses

The recordings took place in a dimly lit and electrically shielded room. Bipolar EMG recordings were made from the upper trapezius descendens (neck), the extensor carpi ulnaris (the wrist extensors in the dorsal posterior forearm), the erector spinae longissimus (lower back), and the peroneus longus (calf). The erector spinae longissimus in the lower back extends the trunk and is activated by backwards leaning or returning to an upright position after flexion. The peroneus longus in the calves is used in eversion of the foot and planar flexion of the ankle, or pushing off the foot. The location of each electrode pair was carefully established according to the SENIAM recommendations for the trapezius, erector spinae longissimus and the peroneus longus (Hermens and Freriks, 1997). To measure the extensor carpi ulnaris activity, the electrodes were placed on a line between the olecranon process of the ulna and the wrist, on the bulky mass of the muscle, approximately 6 cm from the olecranon process. A schematic overview of muscle and electrode locations can be found in **Figures 5**, **6**, but see the SENIAM recommendations for exact electrode placements (Hermens and Freriks, 1997). The electrode sites were cleaned with alcohol and flat-type active electrodes (2 mm diameter), filled with conductive gel, were placed on each muscle with an inter-electrode distance of 20 mm. Two electrodes placed on the C7 vertebrae of the participant served as reference (Common Mode Sense) and ground (Driven Right Leg) electrodes. EMG data was digitized at a rate of 2048 Hz (BioSemi ActiveTwo, Amsterdam, Netherlands). To reduce subject awareness of the aim of the study, the participants were told that the electrodes measured heart rate and skin conductance. The researcher could see and hear the participant through a camera and an intercom.

The data were processed offline using BrainVision Analyzer 2.0 (Brain Products). Eight channels were created, one for each recorded muscle bilaterally, by subtracting electrode pairs. These channels were filtered using a band-pass filter (20–500 Hz, 48 dB/octave) and a notch filter (50 Hz, 48 dB/octave). The signal can be contaminated by the ECG signal. If this ECG contamination is stronger in one electrode of a pair, the ECG noise is not removed by subtraction. This is usually the case in the lower back recordings, as one electrode is closer to the heart than the other. In these cases, an independent component analysis was performed, which produces a sets of independent components present in the data, after which the EMG signal was rebuilt without the ECG component (Mak et al., 2010; Taelman et al., 2011). The data was then rectified, smoothed with a low-pass filter of 50 Hz (48 dB/octave) and segmented into 10 one-second epochs including a 1 s pre-stimulus baseline. The epochs were visually inspected and outlier trials were manually removed per channel per condition. Trials with movement or artifacts in the pre-stimulus baseline and trials with overt movement during the projection of the stimulus were rejected and removed. The remaining trials of each channel were averaged, filtered with 9 Hz, down sampled to 20 Hz and exported.

### Statistical analyses

Only channels with at least 30 valid trials per emotion condition were kept for analysis. To allow for comparison between participants, the data was normalized by expressing the EMG activity as a proportion to baseline for every muscle and emotion. This proportion was calculated by dividing the average activity of 500 ms bins by the average activity of the 1000 ms pre-stimulus baseline, during which the participant stood in a relaxed stance without any stimulus presentation. A value of one signifies no change, whereas values below one signify deactivation and those above one, activation. This resulted in normalized EMG magnitudes of 16 time points (8 s) in the passive viewing condition and 18 time points (9 s) in the active imitation condition, of which the first six time points are during stimulus presentation. To assess the shape and significance of these EMG time courses for each muscle response to each emotion, multilevel growth models were fitted. These models were built step-by-step, starting with a simple linear model with a fixed intercept and slope, to which quadratic and cubic effects of time and random intercepts and slopes are added. Every step is tested by comparing the −2 Log Likelihood with a chi-square distribution. For a more detailed explanation and justification for this method, see Bagiella et al. (2000); Huis in `t Veld et al. (2014). For the passive viewing experiment, all 16 time points were modeled, including the six time points during stimulus presentation. For the imitation condition, only the time period after the participants started moving was modeled.

## Results

### Expression of anger

The first hypothesis pertains to the question of which muscles are used in the active expression of fearful and angry emotion and thus, the results from experiment 2 will be presented first.

**Figure 3:**
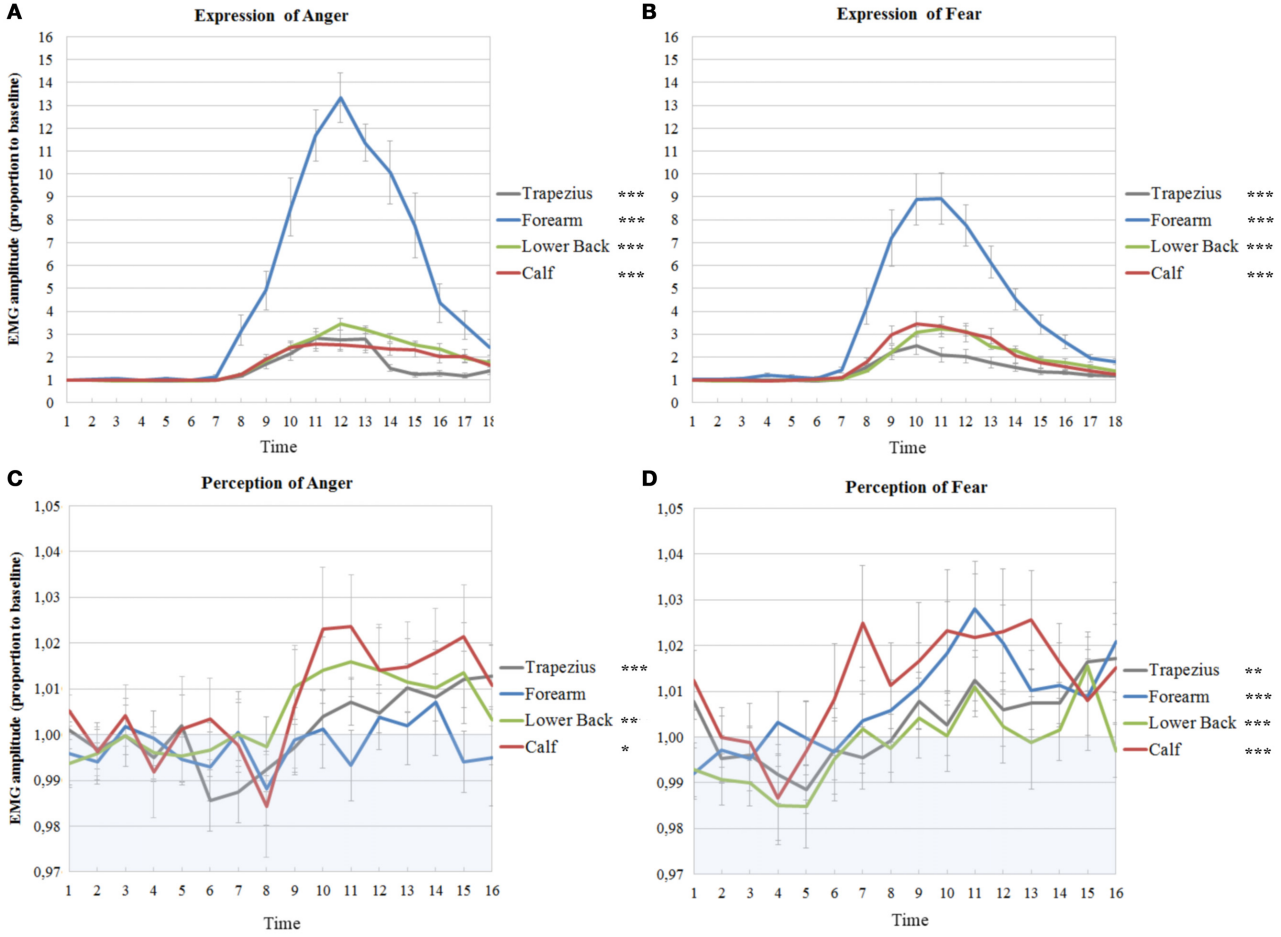
**Muscle activations in experiment 1 and 2 on the right side of the body**. EMG amplitudes and standard errors of the mean of muscles on the right during active imitation of anger **(A)** and fear **(B)** and during passive viewing of anger **(C)** and fear **(D)**. The first six time points represent EMG activity during stimulus presentation. Activity below one (in blue area) indicates deactivation as compared to baseline. Significance levels of the fixed effect of time; ^***^p < 0.001, ^**^p < 0.01, ^*^p < 0.05.

All models of time courses of EMG activity in the active anger condition for the muscles on the right benefited from the inclusion of random intercepts, even though the parameters themselves were not always significant (trapezius; Wald *Z* = 2.93, *p* = 0.003, forearm; Wald *Z* = 1.56, *p* > 0.05, lower back; Wald *Z* = 1.42, *p* > 0.05, calf; Wald *Z* = 1.38, *p* > 0.05). The EMG time courses of the right trapezius, the right forearm and the right calf were best described by a model with a fixed quadratic effect of time [trapezius; *F*_(1, 255)_ = 72.78, *p* < 0.001, forearm; *F*_(1, 252)_ = 205.23, *p* < 0.001, calf; *F*_(1, 237)_ = 116.22, *p* < 0.001], with an additional random effect of time for the forearm (Wald *Z* = 1.86, *p* = 0.06) and the calf (Wald *Z* = 2.59, *p* = 0.01). A model with a fixed cubic effect of time [*F*_(1, 252)_ = 4.51, *p* = 0.035] and a random effect of time (Wald *Z* = 2.04, *p* = 0.04) best fitted the EMG time course of the right lower back (see Table 1A and Figure 3A).

**Table 1:** **Expression of anger**.

	***N***		**Fixed parameter**	***SE b***	**95% CI**	**(U)**	**Random parameter**	**SE Var**
			***b***		**(L)**		**Variance**	
**(A) PARAMETER ESTIMATES OF THE FIXED AND RANDOM EFFECTS IN THE ANGER IMITATION CONDITION OF MUSCLES ON THE RIGHT**								
Trapezius	23	Intercept	−3.988	0.697	−5.360	−2.615	0.670	0.229
		Linear	1.039	0.120	0.802	1.275^***^		
		Quadratic	−0.042	0.005	−0.052	−0.0326^***^		
Forearm	24	Intercept	−34.598	2.938	−40.382	−28.814	16.199	10.370
		Linear	7.319	0.506	6.323	8.315^***^	0.153	0.082
		Quadratic	−0.296	0.021	−0.337	−0.255^***^		
Lower back	23	Intercept	−2.462	1.571	−5.557	0.632	0.332	0.233
		Linear	0.424	0.433	−0.430	1.279	0.004	0.002
		Quadratic	0.028	0.038	−0.046	0.103		
		Cubic	−0.002	0.001	−0.004	−0.0002^*^		
Calf	22	Intercept	−3.381	0.448	−4.263	−2.499	0.304	0.221
		Linear	0.896	0.080	0.739	1.053^***^	0.010	0.004
		Quadratic	−0.034	0.003	−0.041	−0.028^***^		
**(B) PARAMETER ESTIMATES OF THE FIXED AND RANDOM EFFECTS IN THE ANGER IMITATION CONDITION OF MUSCLES ON THE LEFT**								
Trapezius	24	Intercept	−2.691	0.388	−3.454	−1.928	0.218	0.158
		Linear	0.750	0.068	0.615	0.884^***^	0.006	0.002
		Quadratic	−0.029	0.003	−0.034	−0.024^***^		
Forearm	23	Intercept	−30.766	2.580	−35.844	−25.688	1.258	0.766
		Linear	6.515	0.456	5.618	7.412^***^		
		Quadratic	−0.263	0.019	−0.300	−0.226^***^		
Lower back	23	Intercept	−5.505	0.483	−6.456	−4.555	0.374	0.258
		Linear	1.302	0.084	1.137	1.468^***^	0.005	0.002
		Quadratic	−0.050	0.003	−0.057	−0.044^***^		
Calf	22	Intercept	−2.946	0.424	−3.781	−2.111	0.266	0.184
		Linear	0.802	0.074	0.656	0.948^***^	0.005	0.002
		Quadratic	−0.031	0.003	−0.037	−0.025^***^		

Parameter estimates, 95% confidence intervals and significance levels of the best fitting model for each muscle on the (A) right and (B) left in the anger imitation condition.

* p < 0.05;

*** *p < 0.001*.

**Figure 4:**
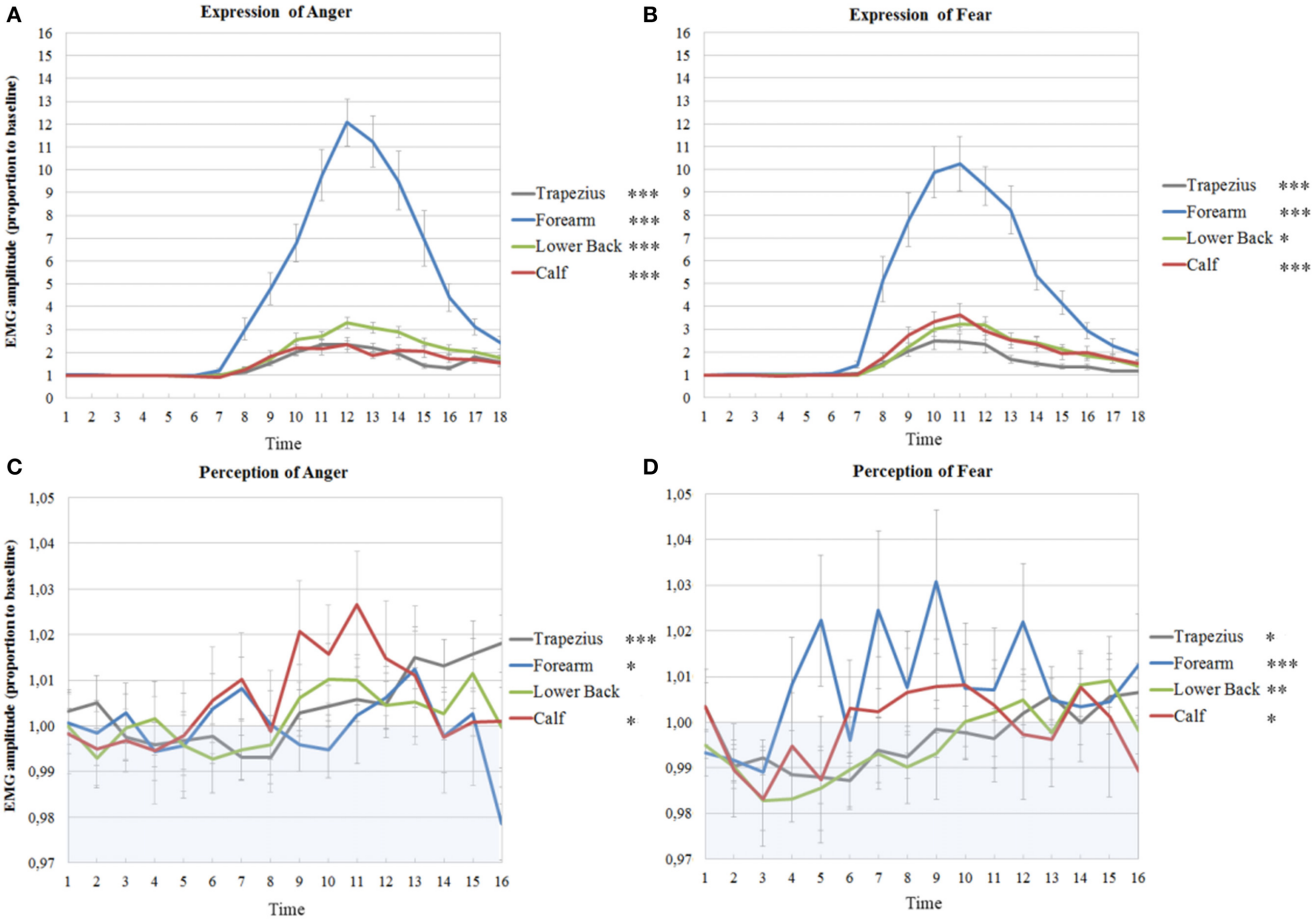
**Muscle activations in experiment 1 and 2 on the left side of the body**. EMG amplitudes and standard errors of the mean of muscles on the left during active imitation of anger **(A)** and fear **(B)** and during passive viewing of anger **(C)** and fear **(D)**. The first six time points represent EMG activity during stimulus presentation. Activity below one (in blue area) indicates deactivation as compared to baseline. Significance levels of the fixed effect of time; ^***^p < 0.001, ^**^p < 0.01, ^*^p < 0.05.

On the left, the random intercepts did not reach significance but inclusion did improve the models (trapezius; Wald *Z* = 1.38, *p* > 0.05, forearm; Wald *Z* = 1.65, *p* > 0.05, lower back; Wald *Z* = 1.45, *p* > 0.05, calf; Wald *Z* = 1.45, *p* > 0.05). Trapezius, forearm, lower back and calf EMG time courses were best described by models with a fixed quadratic effect of time [trapezius; *F*_(1, 249)_ = 111.69, *p* < 0.001, forearm; *F*_(1, 271)_ = 194.97, *p* < 0.001, lower back; *F*_(1, 245)_ = 216.19, *p* < 0.001; calf; *F*_(1, 237)_ = 108.40, *p* < 0.001] and with an additional random effect of time (trapezius; Wald *Z* = 2.48, *p* = 0.013, lower back; Wald *Z* = 1.99, *p* = 0.047, calf; Wald *Z* = 2.23, *p* = 0.025) (see Table 1B and Figure 4A).

### Expression of fear

Random intercepts were also included in all models on the right (trapezius; Wald *Z* = 2.93, *p* = 0.003, forearm; Wald *Z* = 2.38, *p* = 0.017, lower back; Wald *Z* = 1.24, *p* > 0.05, calf; Wald *Z* = 3.01, *p* = 0.003). EMG activity of the right trapezius, forearm and calf were found to follow a fixed cubic trend, unlike the quadratic trends found for anger expression [trapezius; *F*_(1, 253)_ = 22.01, *p* < 0.001, forearm; *F*_(1, 265)_ = 29.63, *p* < 0.001, calf; *F*_(1, 246)_ = 22.69, *p* < 0.001]. Activity in the lower back also followed a fixed cubic effect of time [*F*_(1, 248)_ = 14.50, *p* < 0.001] where the slopes vary across participants (Wald *Z* = 1.86, *p* = 0.06), which was also found for anger expression (see Table 2A and Figure 3B).

**Table 2:** **Expression of fear**.

	***N***		**Fixed parameter**	***SE b***	**95% CI**	**(U)**	**Random parameter**	**SE Var**
			***b***		**(L)**		**Variance**	
**(A) PARAMETER ESTIMATES OF THE FIXED AND RANDOM EFFECTS IN THE FEAR IMITATION CONDITION OF MUSCLES ON THE RIGHT**								
Trapezius	22	Intercept	−9.720	1.674	−13.017	−6.422	0.345	0.118
		Linear	2.873	0.464	1.959	3.787^***^		
		Quadratic	−0.221	0.041	−0.301	−0.141^***^		
		Cubic	0.005	0.001	0.003	0.007^***^		
Forearm	24	Intercept	−52.953	6.826	−66.392	−39.514	3.882	1.631
		Linear	14.341	1.881	10.637	18.045^***^		
		Quadratic	−1.071	0.164	−1.393	−0.749^***^		
		Cubic	0.025	0.005	0.016	0.034^***^		
Lower back	23	Intercept	−10.410	1.491	−13.3463	−7.474	0.238	0.192
		Linear	2.820	0.413	2.006	3.634^***^	0.003	0.002
		Quadratic	−0.189	0.036	−0.260	−0.118^***^		
		Cubic	0.004	0.001	0.002	0.006^***^		
Calf	21	Intercept	−15.298	2.205	−19.640	−10.955	0.987	0.328
		Linear	4.256	0.610	3.054	5.458^***^		
		Quadratic	−0.312	0.053	−0.417	−0.207^***^		
		Cubic	0.007	0.001	0.004	0.010^***^		
**(B) PARAMETER ESTIMATES OF THE FIXED AND RANDOM EFFECTS IN THE FEAR IMITATION CONDITION OF MUSCLES ON THE LEFT**								
Trapezius	23	Intercept	−9.405	1.804	−12.957	−5.853	0.336	0.121
		Linear	2.747	0.501	1.762	3.733^***^		
		Quadratic	−0.206	0.044	−0.292	−0.120^***^		
		Cubic	0.005	0.001	0.002	0.007^***^		
Forearm	21	Intercept	−57.413	7.357	−71.905	−42.921	4.546	1.814
		Linear	15.304	2.029	11.307	19.301^***^		
		Quadratic	−1.113	0.177	−1.461	−0.765^***^		
		Cubic	0.025	0.005	0.015	0.034^***^		
Lower back	22	Intercept	−9.072	1.922	−12.856	−5.288	0.229	0.087
		Linear	2.415	0.533	1.366	3.464^***^		
		Quadratic	−0.151	0.047	−0.243	−0.059^***^		
		Cubic	0.003	0.001	0.0001	0.005^*^		
Calf	23	Intercept	−13.382	2.358	−18.025	−8.740	1.058	0.341
		Linear	3.690	0.651	2.409	4.971^***^		
		Quadratic	−0.263	0.057	−0.374	−0.151^***^		
		Cubic	0.006	0.002	0.003	0.009^***^		

Parameter estimates, 95% confidence intervals and significance levels of the best fitting model for each muscle on the (A) right and (B) left in the fear imitation condition.

* p < 0.05;

*** *p < 0.001*.

On the left, the intercepts varied across participants in all models (trapezius; Wald *Z* = 2.77, *p* = 0.006, forearm; Wald *Z* = 2.51, *p* = 0.012, lower back; Wald *Z* = 2.65, *p* = 0.008, calf; Wald *Z* = 3.10, *p* = 0.002). Furthermore, the time courses were similar as those on the right side, with cubic effects of time [trapezius; *F*_(1, 262)_ = 15.60, *p* < 0.001, forearm; *F*_(1, 239)_ = 25.61, *p* < 0.001, lower back; *F*_(1, 253)_ = 4.44, *p* = 0.036, calf; *F*_(1, 269)_ = 13.41, *p* < 0.001] (see Table 2B and Figure 4B).

### Passive viewing of angry expressions

It was hypothesized that the muscles involved in the active expression of emotion also show activation during passive viewing of these emotions. The results of experiment 1 are shown below.

The intercepts of all time courses of muscles on the right in response to anger showed significant variance across participants (trapezius; Wald *Z* = 2.72, *p* = 0.007, forearm; Wald *Z* = 2.60, *p* = 0.009, lower back; Wald *Z* = 2.22, *p* = 0.026, calf; Wald *Z* = 2.72, *p* = 0.007). Furthermore, the EMG time course of the right trapezius was best described by a model with a fixed quadratic effect of time [*F*_(1, 307)_ = 16.62, *p* < 0.001] where the effect of time was also allowed to vary (Wald *Z* = 2.43, *p* = 0.015). Even though there was a significant variance in intercepts for the EMG time courses of the muscle in the forearm, no significant effects of time could be found. The activity of the lower back followed a cubic trend [*F*_(1, 336)_ = 7.23, *p* = 0.008] and the best fitting model also allowed the effect of time to vary (Wald *Z* = 2.81, *p* = 0.005). Finally, a fixed cubic effect of time [*F*_(1, 253)_ = 6.09, *p* = 0.014] was found for the time course of the calf muscle (see Table 3A and Figure 3C).

**Table 3:** **Passive viewing of angry expressions**.

	***N***		**Fixed parameter**	***SE b***	**95% CI**	**95% CI**	**Random parameter**	**SE Var**
			***b***		**(L)**	**(U)**	**Variance**	
**(A) PARAMETER ESTIMATES OF THE FIXED AND RANDOM EFFECTS IN THE ANGER PASSIVE VIEWING CONDITION OF MUSCLES ON THE RIGHT**								
Trapezius	22	Intercept	1.002	0.005	0.991	1.013	0.0004	0.0001
		Linear	−0.002	0.001	−0.004	−0.0005^*^	2.619e-6	1.079e-6
		Quadratic	5.070e-5	5.070e-5	0.0001	0.0003^***^		
Forearm	16	Intercept	0.996	0.006	0.984	1.008	0.0004	0.0002
Lower back	24	Intercept	1.001	0.008	0.986	1.016	0.0003	0.0001
		Linear	−0.005	0.003	−0.012	0.001	7.948e-6	2.826e-6
		Quadratic	0.001	0.0004	0.0002	0.002^*^		
		Cubic	−4.55e-5	1.69e-5	−7.873e-5	−1.220e-5^***^		
Calf	17	Intercept	1.014	0.012	0.991	1.038	0.0008	0.0003
		Linear	−0.010	0.005	−0.019	−0.001^*^		
		Quadratic	0.002	0.0006	0.0004	0.003^*^		
		Cubic	−6.022e-5	2.441e-5	−0.0001	−1.215e-5^*^		
**(B) PARAMETER ESTIMATES OF THE FIXED AND RANDOM EFFECTS IN THE ANGER PASSIVE VIEWING CONDITION OF MUSCLES ON THE LEFT**								
Trapezius	22	Intercept	1.005	0.005	0.994	1.016	0.0004	2.916e-5
		Linear	−0.003	0.001	−0.005	−0.001^**^	3.224e-6	1.296e-6
		Quadratic	0.0002	5.370e-5	0.0001	0.0003^***^		
Forearm	17	Intercept	1.008	0.009	0.991	1.025	0.0002	9.557e-5
		Linear	−0.006	0.004	−0.014	0.002		
		Quadratic	0.001	0.0005	3.478e-6	0.002^*^		
		Cubic	−4.620e-5	2.03e-5	−8.609e-5	−6.208e-6^*^		
Lower back	23	Intercept	0.994	0.007	0.980	1.009	0.001	0.0003
		Linear	0.0009	0.0006	−0.0004	0.002	6.629e-6	2.708e-6
Calf	13	Intercept	0.984	0.009	0.966	1.001		
		Linear	0.006	0.002	0.001	0.010^**^	1.669e-5	7.481e-6
		Quadratic	−0.0003	0.0001	−0.0005	−8.501e-5^**^		

Parameter estimates, 95% confidence intervals and significance levels of the best fitting model for each muscle on the (A) right and (B) left in the anger passive viewing condition.

* p < 0.05;

** p < 0.01;

*** *p < 0.001*.

Also on the left side, the intercepts of most time courses, with the exception of the calf, showed significant variance across participants (trapezius; Wald *Z* = 2.63, *p* = 0.009, forearm; Wald *Z* = 2.50, *p* = 0.013, lower back; Wald *Z* = 2.74, *p* = 0.006). The same model as found in the right trapezius was also found to fit best, with a fixed quadratic effect of time [*F*_(1, 308)_ = 18.80, *p* < 0.001] and a random effect of time (Wald *Z* = 2.49, *p* = 0.013). In contrast to the lack of activity in the right, the left wrist extensors slightly, but significantly, responded to the perception of anger [fixed cubic effect of time; *F*_(1, 254)_ = 5.18, *p* = 0.024]. In the left lower back, a more complicated model was found, with only a significant random, but not fixed, effect of time (Wald *Z* = 2.45, *p* = 0.014) and a significant covariance between intercepts and slopes (−0.80, Wald *Z* = −8.60, *p* < 0.001), signaling that slopes decrease as intercepts increase. Also, a fixed quadratic effect of time [*F*_(1, 182)_ = 7.77, *p* = 0.006] where the effect of time is also allowed to vary (Wald *Z* = 2,23, *p* = 0.026) fit the data of the calf best (see Table 3B and Figure 4C).

### Passive viewing of fearful expressions

In response to fear, the intercepts of all time courses of muscles on the right also significantly varied across participants (trapezius; Wald *Z* = 2.65, *p* = 0.008, forearm; Wald *Z* = 2.37, *p* = 0.018, lower back; Wald *Z* = 3.01, *p* = 0.003, calf; Wald *Z* = 2.34, *p* = 0.02). A different model was found for the right trapezius in response to fear than to anger; the best fitting model included a fixed cubic effect of time [*F*_(1, 294)_ = 6.94, *p* = 0.009] where the effect of time was also allowed to vary (Wald *Z* = 1.78, *p* = 0.07). The right forearm did respond significantly to the perception of fear, in contrast to the lack of response to anger, with a fixed linear effect of time [*F*_(1, 208)_ = 19.96, *p* < 0.001]. The time course of the right lower back indicates a slight response to fear with a simple fixed linear effect of time [*F*_(1, 340)_ = 16.27, *p* < 0.001]. Similar to the response to anger, a model with a fixed cubic effect of time [*F*_(1, 252)_ = 11.99, *p* = 0.001] but with an additional random effect of time (Wald *Z* = 2.12, *p* = 0.034) fit the time course of the calf (see Table 4A and Figure 3D).

**Table 4:** **Passive viewing of fearful expressions**.

	***N***		**Fixed parameter**	***SE b***	**95% CI**	**(U)**	**Random parameter**	**SE Var**
			***b***		**(L)**		**Variance**	
**(A) PARAMETER ESTIMATES OF THE FIXED AND RANDOM EFFECTS IN THE FEAR PASSIVE VIEWING CONDITION OF MUSCLES ON THE RIGHT**								
Trapezius	21	Intercept	1.011	0.007	0.997	1.024	0.0004	0.0002
		Linear	−0.008	0.003	−0.013	−0.003^***^	1.258e-6	7.065e-7
		Quadratic	0.001	0.0003	0.0004	0.002^***^		
		Cubic	−3.545e-5	1.346e-5	−6.194e-5	−8.956e-6^***^		
Forearm	14	Intercept	0.993	0.006	0.980	1.006	0.0004	0.0002
		Linear	0.002	0.0004	0.001	0.002^***^		
Lower back	23	Intercept	0.986	0.006	0.975	0.997	0.0005	0.0002
		Linear	0.001	0.0003	0.001	0.002^**^		
Calf	18	Intercept	1.014	0.010	0.993	1.035	0.001	0.0003
		Linear	−0.009	0.004	−0.017	−0.001^*^	5.390e-6	2.538e-6
		Quadratic	0.002	0.001	0.001	0.003^***^		
		Cubic	−7.415e-5	2.141e-5	−0.0001	−3.198e-5^***^		
**(B) PARAMETER ESTIMATES OF THE FIXED AND RANDOM EFFECTS IN THE FEAR PASSIVE VIEWING CONDITION OF MUSCLES ON THE LEFT**								
Trapezius	22	Intercept	1.007	0.007	0.993	1.021	0.0004	0.0001
		Linear	−0.008	0.003	−0.013	−0.002^***^	4.823e-6	1.820e-6
		Quadratic	0.001	0.0004	0.0003	0.002^***^		
		Cubic	−3.480e-5	1.432e-5	−6.299e-5	−6.615e-6^**^		
Forearm	12	Intercept	0.985	0.010	0.964	1.005	0.001	0.0003
		Linear	0.007	0.002	0.004	0.011^***^		
		Quadratic	−0.0004	9.819e-5	−0.001	−0.0002^***^		
Lower back	22	Intercept	0.999	0.007	0.985	1.013	0.0004	0.0001
		Linear	−0.008	0.0035	−0.014	−0.002^**^	6.322e-6	2.614e-6
		Quadratic	0.001	0.0004	0.0004	0.002^***^		
		Cubic	−4.864e-5	1.586e-5	−7.986e-5	−1.742e-5^***^		
Calf	15	Intercept	1.000	0.012	0.976	1.025	0.001	0.0004
		Linear	−0.006	0.005	−0.015	0.003	1.298e-5	5.628e-6
		Quadratic	0.001	0.0006	2.343e-5	0.002^*^		
		Cubic	−5.645e-5	2.337e-5	−0.0001	−1.037e-5^**^		

Parameter estimates, 95% confidence intervals and significance levels of the best fitting model for each muscle on the (A) right and (B) left in the fear passive viewing condition.

* p < 0.05;

** p < 0.01;

*** *p < 0.001*.

On the left side of the body, the intercepts of all models also varied significantly (trapezius; Wald *Z* = 2.56, *p* = 0.011, forearm; Wald *Z* = 2.36, *p* = 0.018, lower back; Wald *Z* = 2.20, *p* = 0.028, calf; Wald *Z* = 2.29, *p* = 0.022). EMG amplitudes of the left trapezius followed the same pattern as on the right, with a fixed cubic effect of time [*F*_(1, 308)_ = 5.90, *p* = 0.016] with varying slopes across participants (Wald *Z* = 2.65, *p* = 0.008). The left forearm also significantly responded to fear, with a fixed cubic effect of time [*F*_(1, 183)_ = 14.17, *p* < 0.001] but this model only fit 12 participants. One participant was removed from the analyses on the time courses of the left lower back in response to fear, due to a severely deviant time course. The resulting model included a cubic fixed effect [*F*_(1, 301)_ = 9.4, *p* = 0.002] and a random effect of time (Wald *Z* = 2.42, *p* = 0.016). Finally, the same model as found in the right calf was found in the left calf in response to fear, with a fixed cubic effect of time [*F*_(1, 202)_ = 5.83, *p* = 0.017] but with an additional random effect of time (Wald *Z* = 2.31, *p* = 0.021) (see Table 4B and Figure 4D).

## Discussion

**Figure 5:**
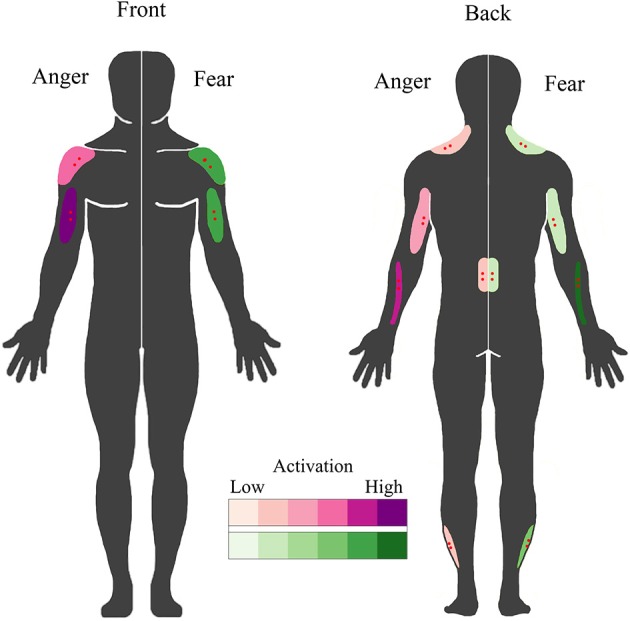
**BACS of emotional expression**. Schematic overview of the muscles involved in overt fearful and angry emotional expression. Muscles involved in the expression of anger are plotted on the left in purple; those involved in fear expression are plotted on the right in green. Higher color intensity means a greater involvement. Electrode locations are shown in red.

The objective of this study was to extend the BACS (Huis in `t Veld et al., 2014) by assessing the involvement of muscles in the forearms, the lower back and the calves in expressing angry and fearful bodily expressions and additionally, to determine whether these muscles also respond to the observers' passive perception of emotion. It was found that the wrist extensors in the forearm (extensor carpi ulnaris) are very strongly involved in angry and, to a lesser extent, fearful movements. Muscles in the lower back (erector spinae longissimus) were activated equally during the expression of both fear and anger, while the muscles in the calf (peroneus longus) were recruited slightly more for the expression of fear. For the muscles in the neck (upper trapezius descendens), the results from BACS I were replicated, with almost overlapping time courses for fear expression and a similar, but slightly delayed, activation pattern for anger expression. When these results are combined with those of BACS I, it is possible to extract unique patterns of muscle activity for angry versus fearful movements. The biceps were found to be the most important muscle for anger expression, followed secondly by forearm, then by shoulder and lastly by triceps activation. In contrast, a fearful expression was marked by forearm activity, followed by an equally strong involvement of shoulders and biceps, while activity of the triceps is quite low (see Figure 5). Considering the function of the muscles, these findings are in line with previous descriptions of angry and fearful movements, such as raising the forearms to the trunk or stretching the arms downward with palms held up (anger) and lifting the arms with the hands held up protectively with the palms outward (fear) (De Meijer, 1989; Wallbott, 1998; Sawada et al., 2003; Coulson, 2004; Kleinsmith et al., 2006; Kessous et al., 2010; Dael et al., 2012b). See Figure 1 for examples of these movements in the stimuli. Furthermore, similar postures were found to correspond to higher arousal ratings (Kleinsmith et al., 2011).

**Figure 6:**
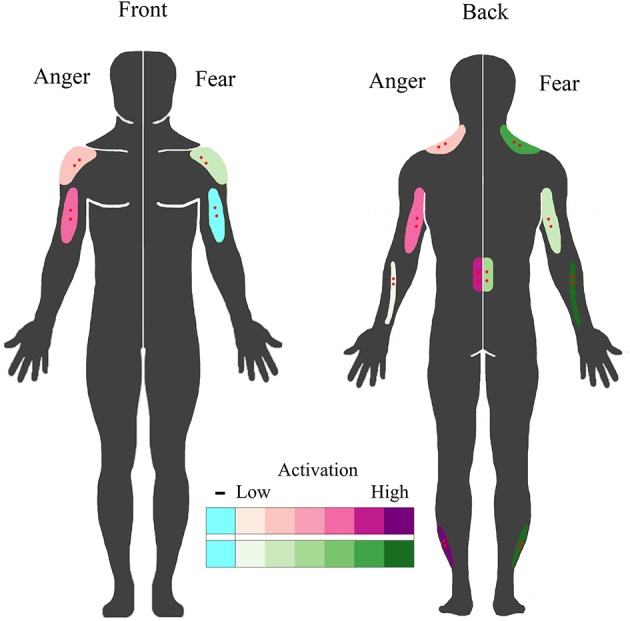
**BACS of emotional perception**. Schematic overview of muscles that covertly respond to the perception of fearful and angry emotional bodily expressions. Muscles that responded to the perception of anger are plotted on the left in purple; those that responded to the perception of fear are plotted on the right in green. Higher color intensity means larger responses, blue indicates deactivation. Electrode locations are shown in red.

The second aim of the study was to assess automatic muscle activations in response to the perception of emotion in others, without any overt movement. The trapezius showed a very similar response both in time (with a quadratic time course for anger and a cubic effect of time for fear) and amplitude as found previously (Huis in `t Veld et al., 2014). The wrist extensors, the most active muscles during fear expression, quite strongly respond only to the perception of fear in others. In contrast, the perception of an angry expression caused a sudden activation of a muscle in the lower back, normally used for leaning backwards. The muscles in the calf strongly activated in response to both fear and anger, but with very different time patterns. Whereas the response of the calf to an angry person only occurs after the angry movement is directed at the observer (i.e., after stimulus presentation), there is an immediate response when a fearful person is seen (during stimulus presentation). The peroneus longus in the calf aids in pushing off the foot in order to walk or run, and thus this pattern may not be surprising, as it may be imperative to immediately avoid anything that presumable made the other person fearful. In contrast, responding to anger is most relevant when it is directed at the observer (Grezes et al., 2013). Fearful bodily expressions very quickly engage action preparation networks and adaptive response strategies, even without visual awareness or attention (Tamietto et al., 2007, 2009; Pichon et al., 2012). A similar interaction between the self-relevance of a perceived emotion and the response can be found between eye gaze direction and emotion recognition, where angry facial expressions are easier to recognize with direct eye contact, whereas the opposite is often true for fear (Wieser and Brosch, 2012). In addition, the fact that some of the largest covert activations can be found in the calves, wrist extensors and the lower back are partly in line with previous studies that showed differential activity of these muscles in response to negative stimuli (Coombes et al., 2006, 2009) or pain or fear for pain (Watson et al., 1997; Geisser et al., 2004; Lelard et al., 2013). Similarly, previous studies have found that it is easier to take a step toward a pleasant stimulus and away from an unpleasant one (Chen and Bargh, 1999; Marsh et al., 2005). Furthermore, as the lower back muscle is involved in moving the torso, this might be related to approach-avoidance tendencies (Azevedo et al., 2005; Horslen and Carpenter, 2011; Eerland et al., 2012) In short, these results, taken together with those described in BACS I, indicate that responses to fear and anger can indeed be distinguished by calf, lower back, trapezius, forearm and biceps (de)activation (see Figure 6).

Furthermore, our findings indicate that not all muscles in the body simply mimic seen behaviors, in contrast to emotional mimicry theories based on facial expressions (Dimberg, 1982). Some muscles very actively involved in movement, like the biceps (Huis in `t Veld et al., 2014) and wrist extensors, activate in response to the perception of the emotion they are most strongly involved in. In contrast, the activity of postural muscles such as in the lower back, neck and calves, is not large during movement and the covert responses seem more reactive. This reaction might be enhanced by the interactive nature of the videos, in contrast to the more traditional facial EMG studies in which photographs are shown. Another indication that these processes may be more complex than mimicry or motor mapping, are the findings that facial muscle responses also occur without conscious awareness (Tamietto et al., 2009) and in response to non-facial stimuli such as bodies and voices (Hietanen et al., 1998; Bradley and Lang, 2000; Magnee et al., 2007; Grezes et al., 2013; Kret et al., 2013). Furthermore, most muscles deactivate during the actual perception of an emotional behavior of another person (during stimulus presentation). These deactivations may be a function of suppression of specific cells in the primary motor cortex, possibly through the supplementary motor area during action observation, to prevent imitation (Bien et al., 2009; Kraskov et al., 2009; Mukamel et al., 2010; Vigneswaran et al., 2013). Stamos et al. (2010) found that action observation inhibited metabolic activity in the area of the spinal cord that contains the forelimb innervations in macaques. In fact, it is known from studies assessing posture that the body can freeze upon the perception of aversive and arousing stimuli (Facchinetti et al., 2006; Roelofs et al., 2010; Stins et al., 2011; Lelard et al., 2013). Other recent studies also support the view that it may be more likely that unconscious emotional muscle responses reflect complex interactive and reactive processes (see Hess and Fischer, 2013 for a review).

The BACS now features descriptions of seven muscles during the expression and perception of anger and fear. In order to generalize the BACS it is important to assess the responses of these muscles in relation to other emotions and a neutral condition. As described in Huis in `t Veld et al. (2014), the stimuli used in the present study did not include neutral stimuli containing the same level of movement as their emotional counterparts. It was decided to first expand the BACS by exploring additional muscles, instead of more emotions, as unique muscle activity patterns are difficult to establish with only four muscles. A follow up experiment, including freely expressed emotions and two control conditions, is currently in preparation. Furthermore, future studies will relate the EMG signal to descriptions of the expressed movement in time and 3D space by using EMG and Motion Capture techniques in concordance. Similar experiments in which participants imitate fixed expressions from video might be improved by using other coding systems such as the Body Action and Posture coding system (BAP; Dael et al., 2012a) or autoBAP (Velloso et al., 2013). Performing principal component analyses (Bosco, 2010) or calculating which muscles co-contract during the expression of which emotions (Kellis et al., 2003), may be suitable methods of statistically appraising which action units are featured for which emotional expressions. Combining these techniques may provide a more complete picture of the specific dynamics of body language and the important contributions of bodily muscles.

Even though the different techniques and their respective coding systems all highlight a different aspect of emotional bodily expressions, one of the advantages of surface EMG is the relative ease of use and the availability of affordable recording systems. The action units in the arms and shoulders, such as the wrist extensors, biceps, triceps and anterior deltoids, are easy to locate and measure and provide very strong movement signals. Assessing their activity patterns in comparison to each other enables the discrimination between angry or fearful movements. In addition, the electrode locations of these action units are also very convenient if one uses wireless electrodes or electrodes in a shirt, making it ideal for use in non-laboratory settings, for example classrooms, hospitals or virtual reality environments.

Most importantly, only EMG is able to measure responses invisible to the naked eye. Future studies interested in assessing these covert activations are encouraged to take the following issues into account. Firstly, the number of trials should be high enough, as some muscles like the wrist extensor and calf muscles were quite sensitive to outliers. Also, EMG activations were best measured from muscles on the right. Implementing affective state information through measuring automatic and non-conscious, unintentional muscle activity patterns may serve as input for previously mentioned human-computer or human-robot interfaces, for example gaming consoles or online learning environments, in order to improve a successful user interaction. Virtual reality especially may prove to be a more effective way than presenting videos to more naturally induce emotion, or even create social interactions, in an immersive but still controlled setting. Furthermore, the BACS can be used as the FACS (Ekman and Friesen, 1978), for example in the study of emotional expression and contagious responses in different cultures (Jack et al., 2012), autism (Rozga et al., 2013), schizophrenia (Sestito et al., 2013), borderline personality (Matzke et al., 2014), aggression or behavioral disorders (Bons et al., 2013; Deschamps et al., 2014), or unconscious pain related behavior (van der Hulst et al., 2010; Aung et al., 2013), just to name a few.

### Conflict of interest statement

The authors declare that the research was conducted in the absence of any commercial or financial relationships that could be construed as a potential conflict of interest.
